# Supplementary material for: Twin modelling reveals partly distinct genetic pathways to music enjoyment
Source: Nat Commun. 2025 Mar 25;16:2904. doi: 10.1038/s41467-025-58123-8 (PMC11937235; doi:10.1038/s41467-025-58123-8)
Supplement: Supplementary file 1 — Supplementary Information [file 41467_2025_58123_MOESM1_ESM.pdf]

Supplementary information for *Twin modelling reveals partly distinct genetic pathways to music enjoyment*

Table of Contents

SUPPLEMENTARY NOTE 1 2
SUPPLEMENTARY NOTE 2 3
SUPPLEMENTARY NOTE 3 4
SUPPLEMENTARY NOTE 4 5
SUPPLEMENTARY NOTE 5 6
SUPPLEMENTARY NOTE 6 8
SUPPLEMENTARY NOTE 7 9

SUPPLEMENTARY FIGURES 10

SUPPLEMENTARY FIGURE 1 10
SUPPLEMENTARY FIGURE 2 11
SUPPLEMENTARY FIGURE 3 12
SUPPLEMENTARY FIGURE 4 13
SUPPLEMENTARY FIGURE 5 14
SUPPLEMENTARY FIGURE 6 15
SUPPLEMENTARY FIGURE 7 16
SUPPLEMENTARY FIGURE 8 17
SUPPLEMENTARY FIGURE 9 18
SUPPLEMENTARY FIGURE 10 19
SUPPLEMENTARY FIGURE 11 20

SUPPLEMENTARY TABLES 21

SUPPLEMENTARY TABLE 1 21
SUPPLEMENTARY TABLE 2 22
SUPPLEMENTARY TABLE 3 23
SUPPLEMENTARY TABLE 4 24
SUPPLEMENTARY TABLE 5 25
SUPPLEMENTARY TABLE 6 26
SUPPLEMENTARY TABLE 7 27
SUPPLEMENTARY TABLE 8 28

SUPPLEMENTARY REFERENCES 29

Supplementary Note 1. *Test statistics for alternative estimators.* Here, we provide robust test statistics for models for which we used the maximum likelihood estimator with robust ("MLR") sandwich (Huber-white) standard error[1]. More information on how lavaan[2] handles robust test statistics can be found at https://users.ugent.be/~yrosseel/lavaan/lavaan2.pdf. Below, we contrast the estimates obtained from the MLR estimator with the results obtained from the full-information maximum likelihood (ML) estimator used in the main analyses.

- *Baseline univariate model:*
  Comparison between models with and without constrained means across sex. Results for the ML estimator: $\chi^2(\Delta df = 30) = 297.5$, $p < .001$. Results for the MLR estimator: $\chi^2(\Delta df = 30) = 288.6$, $p < .001$. *Comparison between models with and without age effects on the BMRQ total score.* Results for the ML estimator: $\chi^2(\Delta df = 1) = 8.58$, $p = .003$. Results for the MLR estimator: $\chi^2(\Delta df = 1) = 9.09$, $p = .003$. In summary, sex and age effects on BMRQ scores are similar with both estimators (ML and MLR).
- *ADE univariate model:*
  Model comparison between models with and without the A component. Results for the ML estimator: $\chi^2(\Delta df = 1) = 458.49$, $p < .001$; Results for the MLR estimator: $\chi^2(\Delta df = 1) = 534.51$, $p < .001$. Therefore, the conclusion that dropping the A component significantly deteriorates model fit remains unaltered when using the MLR estimator.
- *Distinct factors solution vs shared-genetic factor solution:*
  Model comparison test statistics to infer the absence of a single shared genetic or environmental factor. Results for the ML estimator, hybrid independent genetic pathway model: $\chi^2(\Delta df = 5) = 65.14$, $p < .001$. Results for the MLR estimator: $\chi^2(\Delta df = 5) = 64.74$, $p < .001$. Results for the ML estimator, hybrid independent environmental pathway model: $\chi^2(\Delta df = 5) = 34.12$, $p < .001$. Results for the MLR estimator: $\chi^2(\Delta df = 5) = 31.40$, $p < .001$. In summary, using different estimators, the main findings remained essentially the same.

Supplementary Note 2. *Alternative causal models for the Cholesky decomposition.* In the main text, we note that the variance shared between music perceptual abilities and music reward sensitivity could be mainly due to shared genetic factors acting as common causes. This can be drawn as Supplementary Fig. 10. However, there are additional models that could better describe the causal relationships behind the covariances observed (and implied) in the main text. It is possible for the genetic effects shared between the two traits to not act as a common cause. Instead, one trait could mediate genetic effects on one of the two phenotypes. Supplementary Fig. 10 could guide future research in which alternative causal models, which we could not appropriately assess in this study, could be put to the test. Nevertheless, we note that, as our main interest was to quantify the magnitude of heritability for music reward sensitivity beyond shared genetic effects with music perceptual abilities, our main conclusion holds, regardless of the actual causal structure. The only exception would be under reverse causal models depicted in Supplementary Fig. 10 b, as the heritability of music perceptual abilities would not explain the heritability of music reward sensitivity but vice versa. However, we would like to note that under this latter scenario, our estimates are still conservative estimates of the adjusted heritability of music reward sensitivity.

Supplementary Note 3. *Genetic heterogeneity of facets of music reward is robust to the exclusion of the sensory-motor facet.* Despite no evidence for a shared factor, the genetic correlations between the facets suggest the possibility of multiple shared genetic factors. To explore this possibility, we applied a principal component analysis of the additive genetic correlation matrix extracted from the correlated factor solution[3]. To get a data-driven estimate of the dissimilarity between the genetic factors, we performed a spectral decomposition of the 5 × 5 additive genetic correlation matrix **A**. In Supplementary Fig. 11, we plot the two eigenvectors explaining the majority of genetic variance, PC1 and PC2. As can be seen, the sensorimotor facet was mostly distinct from every other facet. This indicated that the genetic variance associated with the sensory-motor facet displays the smallest overlap with any other facet. Based on the results of the principal component analysis, we performed a new multivariate analysis without the sensory-motor facet, fitting a correlated factor model and a hybrid independent-genetic pathway model to emotion-evocation, mood regulation, music-seeking, and social reward facets data. The comparison between these two models still indicated some genetic heterogeneity across emotion-evocation, mood regulation, musical seeking, and social reward facets (AIC = 230465 and BIC = 230725, AIC = 230503 and BIC = 230749, respectively; $\chi^2(\Delta df = 2) = 42.14$, $p < .001$). This conclusion did not change by using the different MLR estimator ($\chi^2(\Delta df = 2) = 45.38$, $p < .001$). The findings confirm that a single shared-genetic factor model of music reward sensitivity facets is a worse solution than the distinct factor model, even when excluding the most genetically distinct facet.

Supplementary Note 4. *What partly distinct genetic pathways imply for studies of musical anhedonia?* On the one hand, we found no single shared genetic factor underlying inter-individual differences in different facets of music reward sensitivity. On the other hand, we also found moderate genetic correlations between such facets. These results are consistent with moderate to large genetic pleiotropy across different facets of music reward sensitivity and could explain why using music reward sensitivity as a tool to, for example, cluster individuals in different categories from anhedonics to "hyper-hedonics" has been successful as a strategy to link individual differences in music enjoyment to physiological and neurobiological differences[4–7]. Musical anhedonia is a well-studied condition "diagnosed" in individuals with a BMRQ total score less than a certain threshold but without a deficit music perception or general hedonic processes. Previous studies have shown that musical anhedonia relates to physiological and neurobiological differences, notably indicating that individuals with musical anhedonia tend to show altered structural and functional connectivity in cortico-subcortical loops along the auditory ventral stream[5,6,8].

However, our findings suggest that two individuals with the same level of musical reward sensitivity might not necessarily be alike. Given that genetic pathways to different facets of music reward sensitivity are partly distinct, different individuals coexisting in the same musical anhedonia group are likely to carry different DNA variants contributing to the trait. If genetic effects in this study were to influence music reward sensitivity via the brain, then the finding of distinct genetic pathways would imply that previous studies may have missed important mechanisms underlying different manifestations of musical anhedonia as a simple consequence of using BMRQ total scores rather than facet data. By averaging across the facets, prior studies may have enhanced the detection of neurobiological associations with more strongly genetically correlated facets (e.g., genetic variants associated with emotion evocation, mood regulation, and social reward facets) while blunting detection of neurobiological mechanisms related to other facets (e.g., sensory-motor facet).

Such genetic heterogeneity implies that there may be different neural mechanisms for different profiles of musical anhedonia and that different genetic influences may exert their effects on musical anhedonia following different pathways. For example, genetic effects over the sensory-motor facet could be interpreted in light of the differentiation of the roles of the two streams (ventral and dorsal) in different aspects of music cognition, from perception to pleasure[9]. Based on previous models[8–10], one might speculate that genetic variants associated with structural and functional connectivity differences along the dorsal stream could partially capture genetic dissociations like those found in the present study. In contrast, previous averaging across genetically heterogeneous facets could explain why other studies found specific associations between alteration in cortico-subcortical loops along the auditory ventral but not the dorsal stream[5,6]. Further research could put this to the test by comparing genetic correlations between different facets of music reward sensitivity and functional or structural connectivity between selected ventral and dorsal stream areas.

Supplementary Note 5. *Barcelona music reward questionnaire*[11]. The English version of the questionnaire is outlined below. For simplicity, reversed-scored items are indicated by the superscript r. The facets to which items belong are indicated between parentheses.

Instructions:

Each item of this questionnaire is a statement that a person may either agree with or disagree with. For each item, indicate how much you agree or disagree with what the item says. Please respond to all the items; do not leave any blank. Choose only one response to each statement. Please be as accurate and honest as you can be. Respond to each item as if it were the only item. That is, do not worry about being consistent in your responses. Choose from completely disagree (left) to completely agree (right) one of the five options.

Response options:

1;2;3;4;5

completely disagree; disagree; neither agree nor disagree; agree; completely agree.

Items:

1. When I share music with someone I feel a special connection with that person. (Social reward)

2. In my free time I hardly listen to music. ^{r} (Music seeking)

3. I like listen to music that contains emotion. (Emotion evocation)

4. Music keeps me company when I'm alone. (Mood regulation)

5. I don't like to dance, not even with music I like. ^{r} (Sensory motor)

6. Music makes me bond with other people. (Social reward)

7. I inform myself about music I like. (Music seeking)

8. I get emotional listening to certain pieces of music. (Emotion evocation)

9. Music calms and relaxes me. (Mood regulation)

10. Music often makes me dance. (Sensory motor)

11. I'm always looking for new music. (Music seeking)

12. I can become tearful or cry when I listen to a melody that I like very much. (Emotion evocation)

13. I like to sing or play an instrument with other people. (Social reward)

14. Music helps me chill out. (Mood regulation)

15. I can't help humming or singing along to music that I like. (Sensory motor)

16. At a concert I feel connected to the performers and the audience. (Social reward)

17. I spend quite a bit of money on music and related items. (Music seeking)

18. I sometimes feel chills when I hear a melody that I like. (Emotion evocation)

19. Music comforts me. (Mood regulation)

20. When I hear a tune I like a lot I can't help tapping or moving to its beat. (Sensory motor)

The Swedish version of the questionnaire is found below. Please refer to the English version above for details on reversed items and facets.

Instructions:

Varje påstående i detta avsnitt är något man antingen kan hålla med om eller inte hålla med om. För varje påstående vill vi att du indikerar hur mycket du håller med om det. Försök att vara så ärlig du kan när du svarar. Svara på varje påstående som om det vore det enda påståendet, dvs oroa dig inte för att vara inkonsekvent i dina svar.

Response options:

1;2;3;4;5

Håller inte alls med; Håller delvis inte med; Varken eller; Håller delvis med; Håller helt med.

Items:

1. När jag delar en musikupplevelse med någon känner jag ett särskilt band till den personen.
2. På min fritid lyssnar jag sällan på musik.
3. Jag tycker om att lyssna till känslosam musik.
4. Musik håller mig sällskap när jag är ensam.
5. Jag tycker inte om att dansa, inte ens till musik som jag gillar.
6. Musik får mig att knyta an till andra personer.
7. Jag håller mig uppdaterad kring musik jag tycker om.
8. ag blir känslosam när jag lyssnar till vissa musikstycken.
9. Musik gör mig lugn och avslappnad.
10. Musik får mig ofta att dansa.
11. Jag håller alltid utkik efter ny musik.
12. Jag kan få tårar i ögonen eller börja gråta när jag lyssnar till en melodi som jag tycker mycket om.
13. Jag tycker om att sjunga eller spela instrument tillsammans med andra.
14. Musik hjälper mig att ta det lugnt.
15. Jag kan inte låta bli att nynna eller sjunga med i musik som jag gillar.
16. Vid konserter känner jag samhörighet med artisterna och publiken.
17. Jag spenderar en hel del pengar på musik och musikrelaterade saker.
18. Ibland kan jag få rysningar när jag hör en melodi som jag tycker om.
19. Musik tröstar mig.
20. När jag hör en låt som jag tycker mycket om så kan jag inte låta bli att trumma med eller röra mig till takten.

Supplementary Note 6. *Behavioural Approach System Reward Responsiveness*[12,13]. The English and Swedish versions of the items of the reward responsiveness scale are provided below. Reversed-scored items are indicated by the superscript r.

English:

Instructions:

Each item of this questionnaire is a statement that a person may either agree with or disagree with. For each item, indicate how much you agree or disagree with what the item says. Please respond to all the items; do not leave any blank. Choose only one response to each statement. Please be as accurate and honest as you can be. Respond to each item as if it were the only item. That is, don't worry about being "consistent" in your responses. Choose from the following four response options:

Response options:

1;2;3;4

very true for me; somewhat true for me; somewhat false for me; very false for me

Items:

4. When I'm doing well at something I love to keep at it. ^{r}

7. When I get something I want, I feel excited and energized. ^{r}

14. When I see an opportunity for something I like I get excited right away. ^{r}

18. When good things happen to me, it affects me strongly. ^{r}

23. It would excite me to win a contest. ^{r}

Swedish:

Instructions:

Hur jag är Varje påstående i detta avsnitt är något man antingen kan hålla med om eller inte hålla med om. För varje påstående vill vi att du anger hur mycket du håller med om det. Var vänlig svara på alla påståenden och försök att vara så ärlig du kan när du svarar.

Response options:

1;2;3;4

Mycket sant för mig ;Något sant för mig; Något falskt för mig; Mycket falskt för mig)

Items:

4. När jag är bra på något älskar jag att hålla på med det

7. När jag får något jag vill ha, blir jag upprymd och fylld av energi

14. När jag ser en möjlighet att göra/få något jag gillar, blir jag uppspelt på direkten

18. När bra saker händer påverkar det mig mycket starkt

23. Jag skulle bli uppspelt av att vinna en tävling

Supplementary Note 7. *Comparison with results obtained using OpenMx.* Below is a contrast between estimates obtained using (1) lavaan, a five-group model (MZ women, MZ men, DZ women, DZ men, DZ opposite-sex), with the inclusion of age as a manifest covariate (e.g., including age in the variance-covariance matrix) and (2) results of a standard analysis using OpenMx, a two-groups (MZ and DZ only) with the inclusion of age as a definition covariate (scripts available at: https://hermine-maes.squarespace.com/s/oneADEvca-s8hc.pdf).

- *AE versus ADE model comparison.* Results obtained from lavaan, manifest covariate, five groups: $\chi^2(\Delta df = 1) = 1.63$, $p = .20$. Results obtained from OpenMx, definition covariate, four groups: $\chi^2(\Delta df = 1) = 0.31$, $p = .57$.
- *AE versus E model comparison.* Results obtained from lavaan $\chi^2(\Delta df = 1) = 458.49$, $p <.001$**.** Results obtained from OpenMx $\chi^2(\Delta df = 1) = 464.90$, $p <.001$***.***
- *Additive genetic variance and heritability.* Results obtained from lavaan, $h_{\text{twin}}^2 = .54$ (95% CI [.51, .58]); $\sigma_A^2 = 101.98$ (95% CI [93.73, 110.23]) Results obtained from OpenMx $h_{\text{twin}}^2 = .56$; $\sigma_A^2 = 110.03$ (95% CI [100.93, 119.18]).

Supplementary Figures

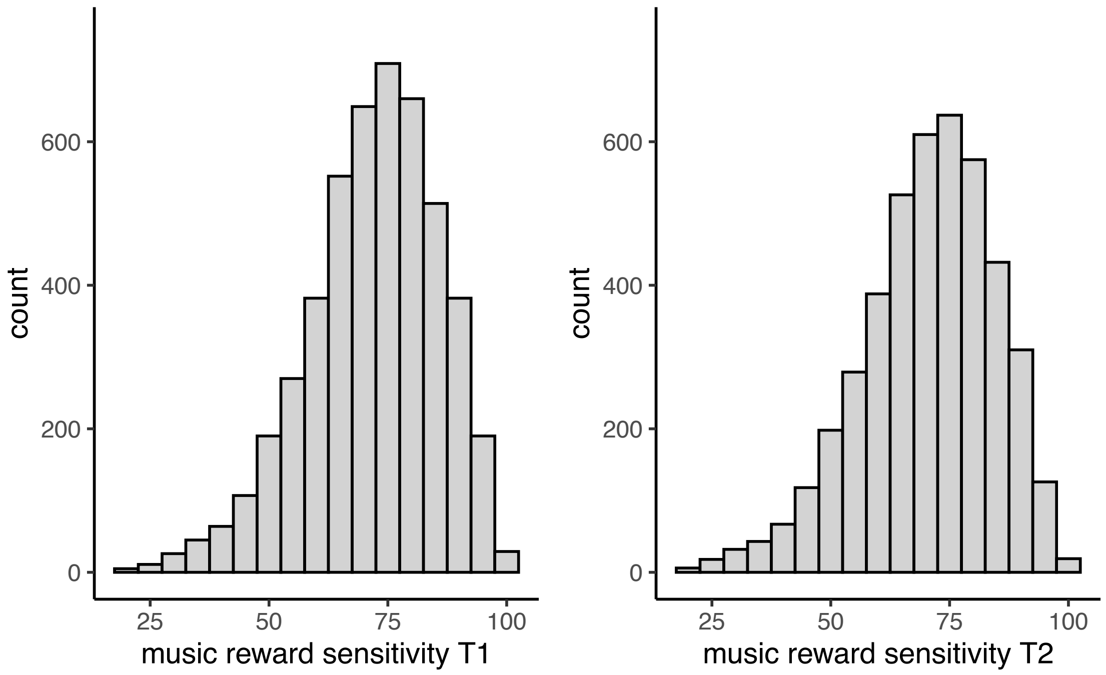

Supplementary Figure 1. *Distribution of BMRQ total scores.* Histogram of music reward sensitivity measured by the BMRQ total score of the Swedish translated version. Each bin spans five BMRQ points on the scale. T1: twin 1; T2: twin 2. Note that the twin order is randomised within pairs, but for opposite-sex dizygotic twins, T1 is always a woman.

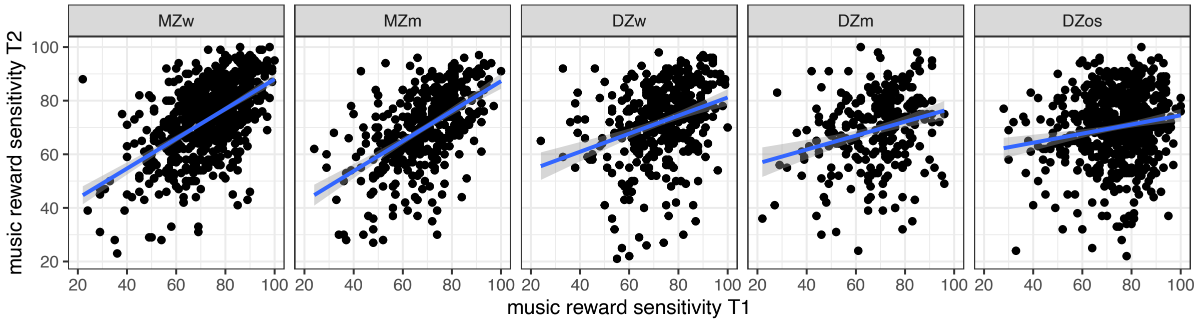

Supplementary Figure 2. Scatter plot of the within-pair BMRQ correlations stratified across zygosity groups. Correlations between twins for music reward sensitivity as measured by the BMRQ total score (Twin 1, T1 and Twin 2, T2). Linear regression lines are shown in blue. Each dot represents a twin pair. MZw: Monozygotic women; MZm: Monozygotic men; DZw: Dizygotic women; DZm: Dizygotic men, DZos: Dizygotic opposite-sex.

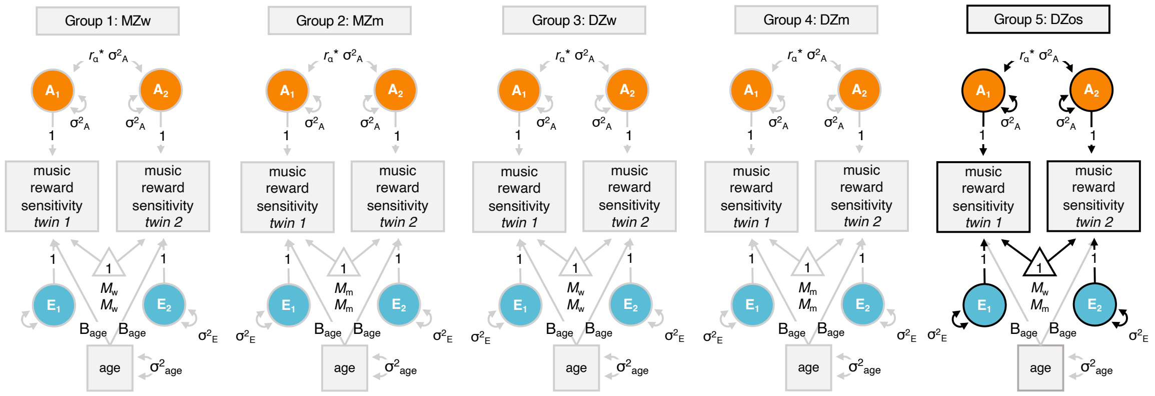

Supplementary Figure 3. *Full AE SEM model.* The final univariate structural equation model is informed by the classical twin design (CTD) and estimated via a direct symmetric approach. In grey, the elements omitted from the simplified graphical representation reported in the main manuscript (Fig. 1; also note that colours match). Each group represents a zygosity. Parameters are constrained to be equal across groups, except for means, which are kept unconstrained across sex. The six parameter estimates can be found in Source Data.

Notes on structural equation models: *Squares or rectangles represent the measured phenotypes; circles are the latent component; double-headed arrows within circles represent the variances associated with the latent components or measured phenotypes; double-headed arrows between circles represent covariances; the triangle represents the phenotypic mean grouped sex;* σ^2*: variance; A: additive genetic component; E: residual environmental component.*

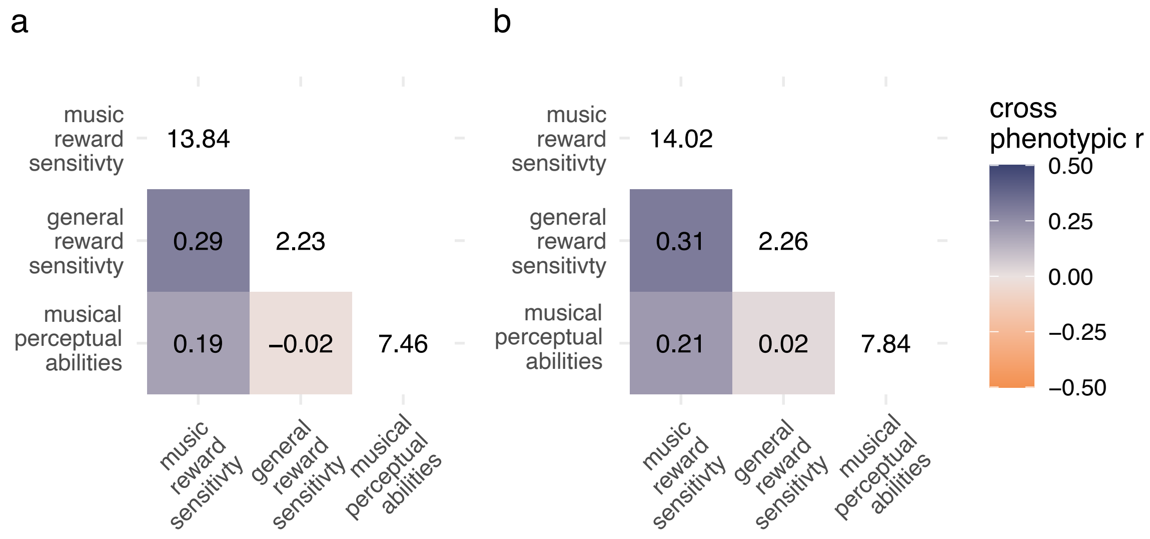

Supplementary Figure 4. *Phenotypic correlations matrix for facets of music reward sensitivity.* Phenotypic correlations and standard deviation (on the diagonal). **a** Estimates obtained from one twin per pair (twin 1). **b** Estimates obtained from the other twins.

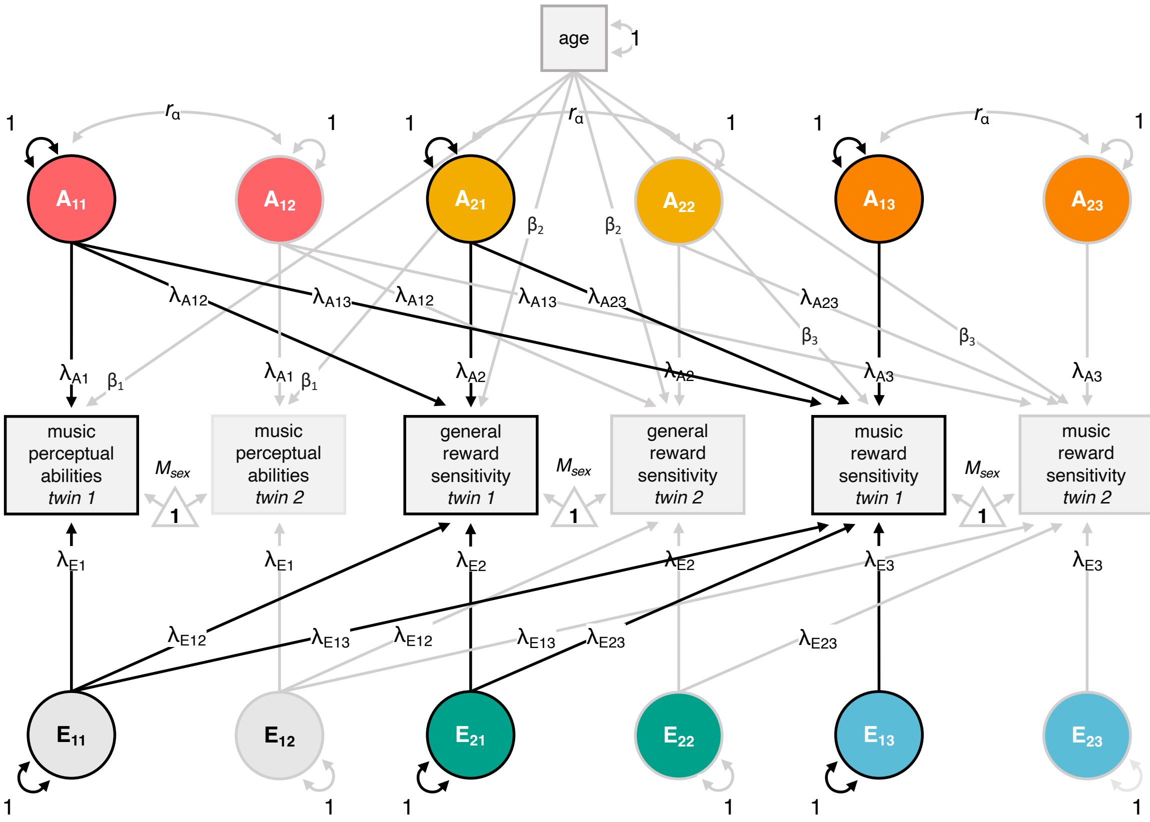

Supplementary Figure 5. *Full AE SEM model.* Cholesky decomposition. For simplicity, only one zygosity group is shown. In grey, the elements omitted from the simplified graphical representation reported in the main manuscript (Fig. 3). Parameters are constrained to be equal across groups, except for means across sex. Note that the colours of the circle match the colour in the main manuscript (Fig. 3), with red corresponding to the additive genetic factor for music perceptual abilities, yellow corresponding to the additive genetic factors for general reward sensitivity, orange corresponding to the additive genetic factor for music reward sensitivity, grey corresponding to the non-common environmental factor for music reward sensitivity, green corresponding to the non-common environmental factor for general reward sensitivity, and turquoise corresponding to the non-common environmental factor for music reward sensitivity. The 21 parameter estimates can be found in the Source Data. (Note that given estimates are standardised, including age as a manifest covariate.)

Notes on structural equation models: one-headed arrow represents regression paths, partitioned in additive genetics (λA) and non-common environmental (λE) paths; dashed one-headed arrows, nonsignificant paths. Other abbreviations and symbols are as in previous Supplementary Figures.

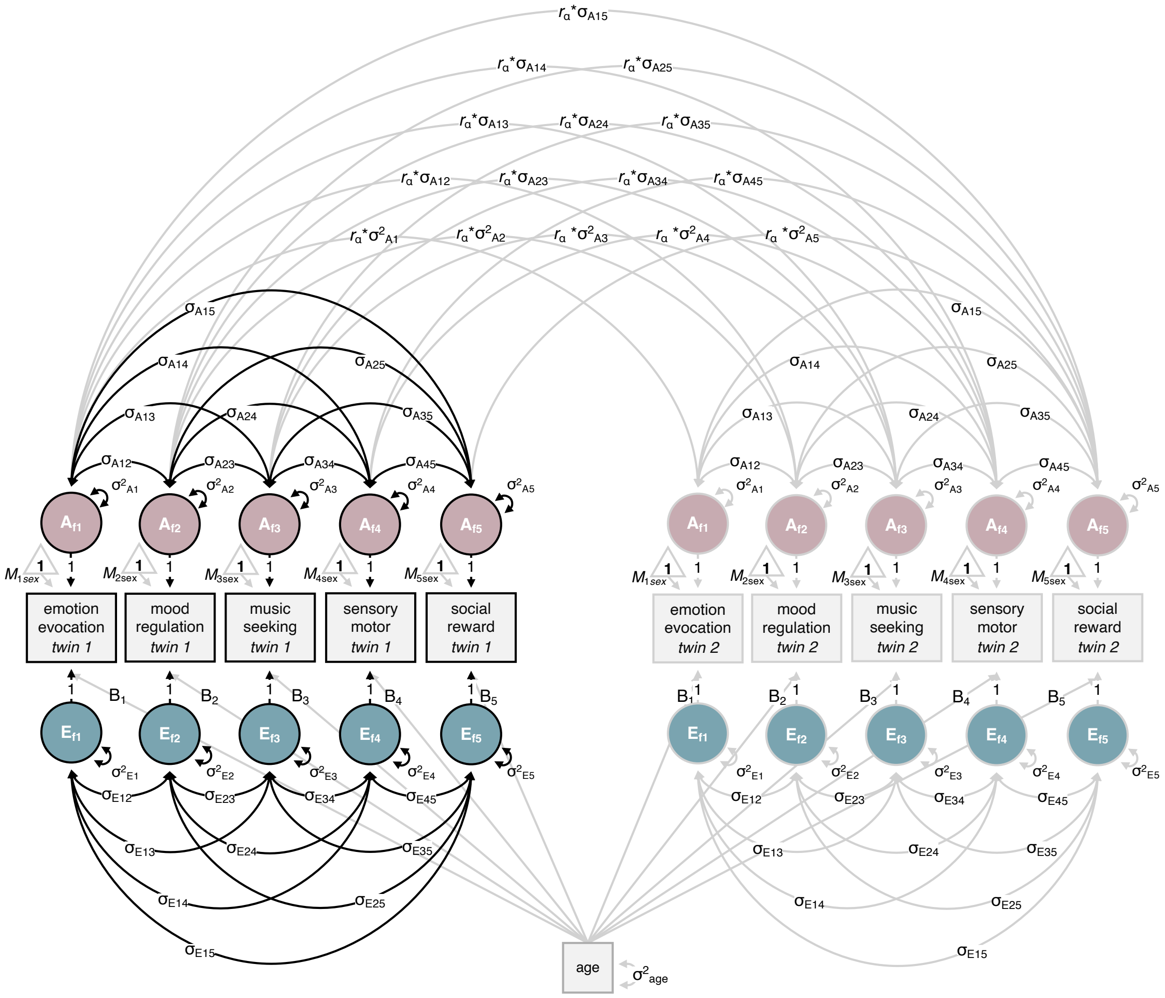

Supplementary Figure 6. *Correlated factor model via direct symmetric approach (distinct factor solution).* To avoid cluttering, only one zygosity group is shown. Variances or covariances are directly estimated. In grey, the elements omitted from the simplified graphical representation reported in the main manuscript (Fig. 5). The 46 parameter estimates can be found in the Source Data.

Notes on structural equation models: σ_{Aij} *represents the additive genetic covariance between* A_f *(f: facet) components i and j. Other abbreviations, symbols, and notes are as in previous Supplementary Figures.*

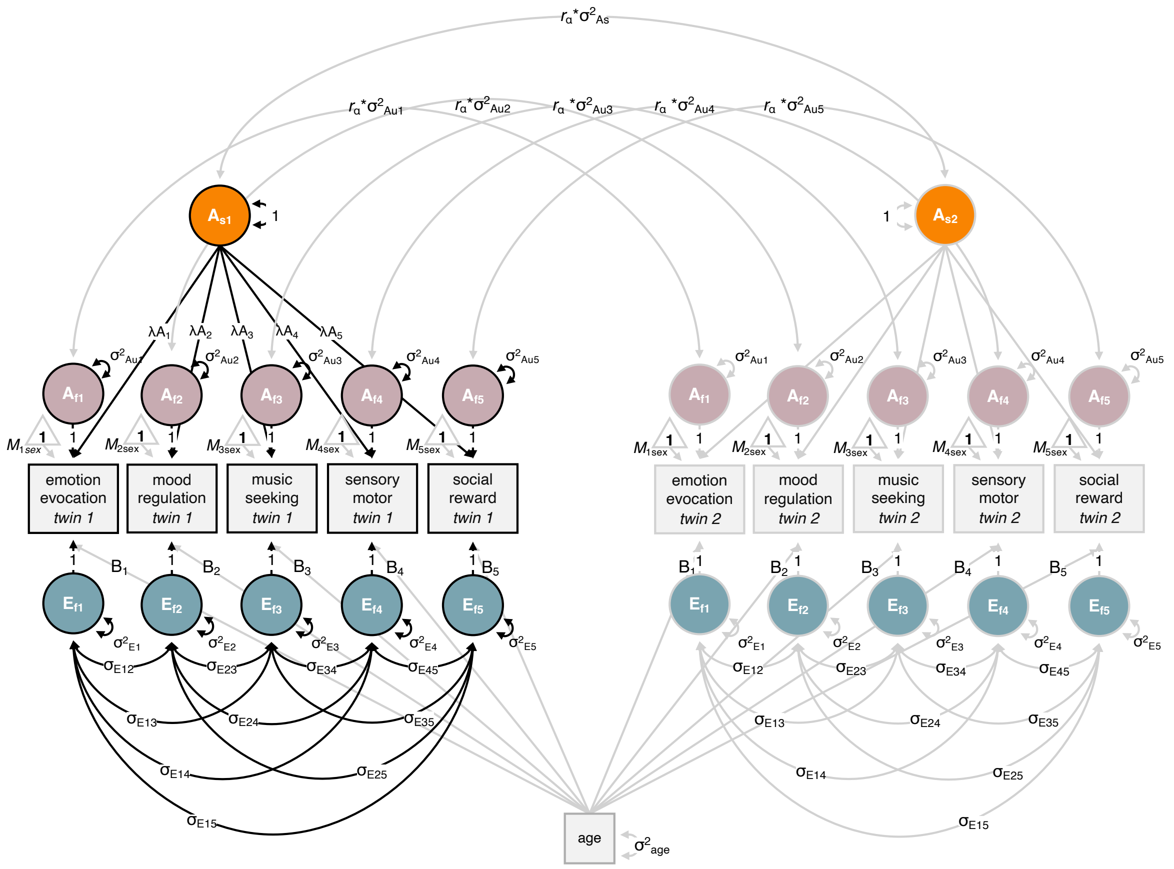

Supplementary Figure 7. *Hybrid Independent pathway model (shared-genetic factor solution).* To avoid cluttering, only one group is shown. Besides the variances of the shared additive genetics (A_s) components, which are fixed to 1, variances or covariances are directly estimated. Note that this model is nested within the model depicted in Supplementary Figure 6. The 41 parameter estimates can be found in the Source Data.

Notes on structural equation models: Here, λ represents the path coefficients from the additive genetic shared factor; s (shared) and u (unique) abbreviations represent shared and unique additive genetic components. Other abbreviations, symbols, and notes are as in previous Supplementary Figures.

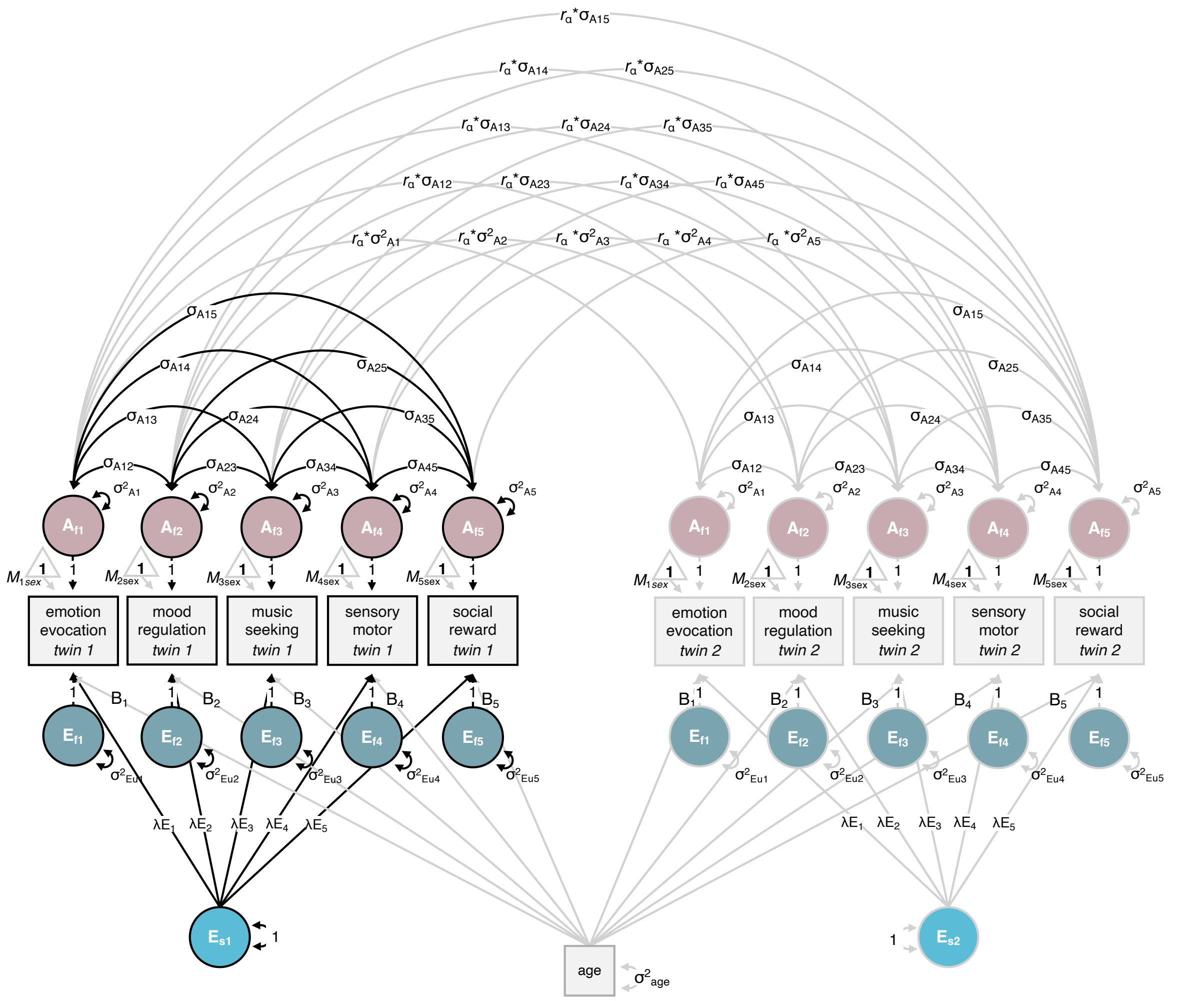

Supplementary Figure 8. *Hybrid Independent pathway model (shared-environmental factor solution).* To avoid cluttering, only one group is shown. Besides the variances of the shared non-common environmental (E_s) components, which are fixed to 1, variances or covariances are directly estimated. Note that this model is nested within the model depicted in Supplementary Figure 6. The 41 parameter estimates can be found in the Source Data.

*Notes on structural equation models: Here, λ represents the path coefficients from the environmental shared factor****.*** *Other abbreviations, symbols, and notes are as in previous Supplementary Figures.*

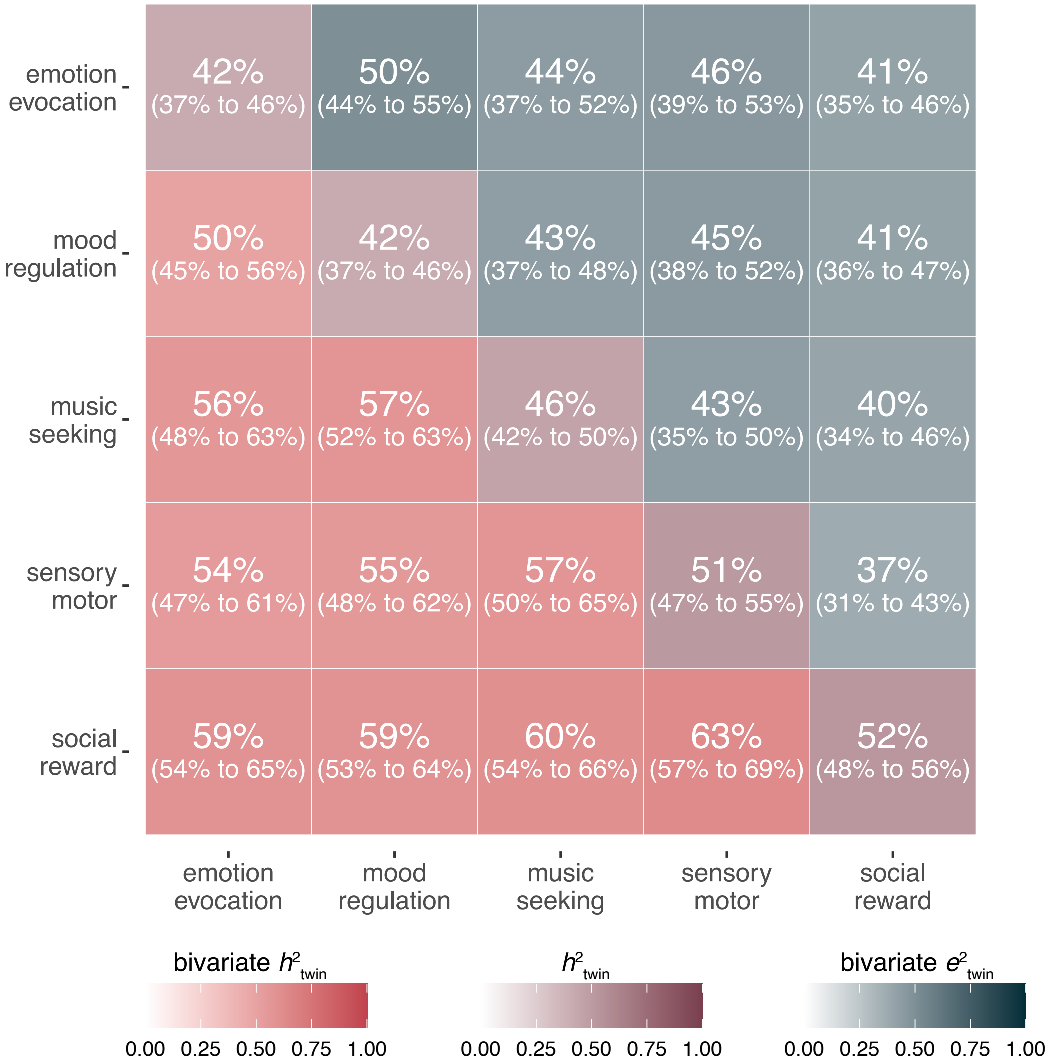

Supplementary Figure 9. *Percentage of BMRQ facet associations explained by genetic and environmental factors.* Below the diagonal is the percentage of phenotypic covariance between any two BMRQ facets accounted for by genetic factors (e.g., bivariate h_{twin}^2). The upper diagonal element represents the percentage of phenotypic covariance accounted for by non-common environmental factors (e.g., bivariate e_{twin}^2). For any two facets, *i* and *j*, the bivariate heritability estimate is obtained as the ratio of the genetic covariance between facets *i* and *j* over the phenotypic covariance between *i* and *j*. Similarly, bivariate estimates for the environmental component are obtained as the ratio of the environmental covariance between facets *i* and *j* over the phenotypic covariance between *i* and *j*. These estimates provide additional information on the association between two phenotypes as they do not correspond to genetic and environmental correlations (e.g., bivariate heritability can be high with low genetic correlations, and vice-versa). Diagonal is h_{twin}^2 estimates, as in the main text. Estimates in parentheses represent 95% CI. bivariate h_{twin}^2: bivariate heritability; bivariate e_{twin}^2: bivariate non-common-environmental.

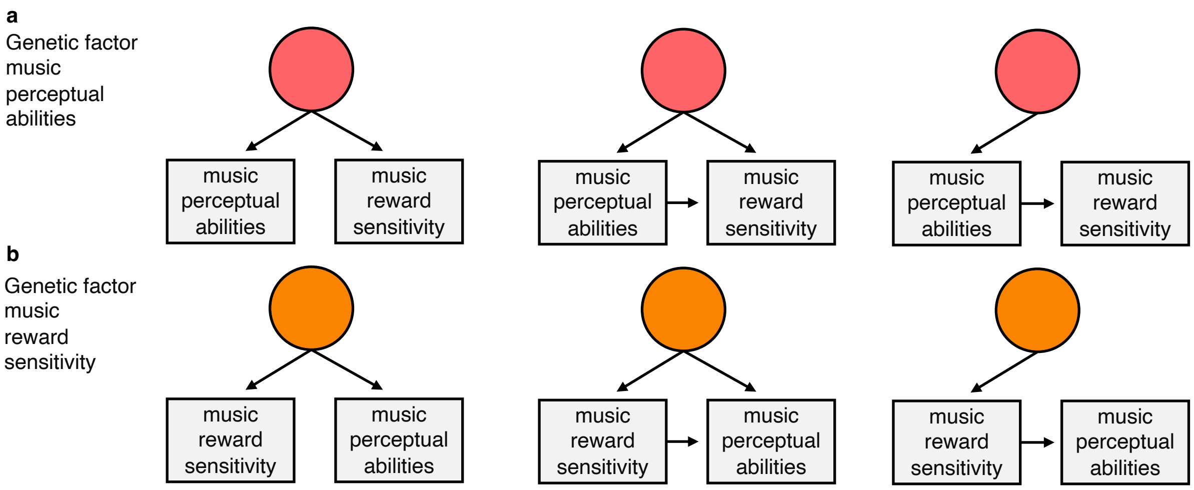

Supplementary Figure 10. *Alternative causal modes for the sequential decomposition.* The observed covariance between musical auditory discrimination and music reward sensitivity can be underpinned by different causal mechanisms. **a** and **b** genetic components associated with music perceptual abilities and with music reward sensitivity. *Notes on structural equation models: Abbreviations, symbols, and notes are as in previous Supplementary Figures.*

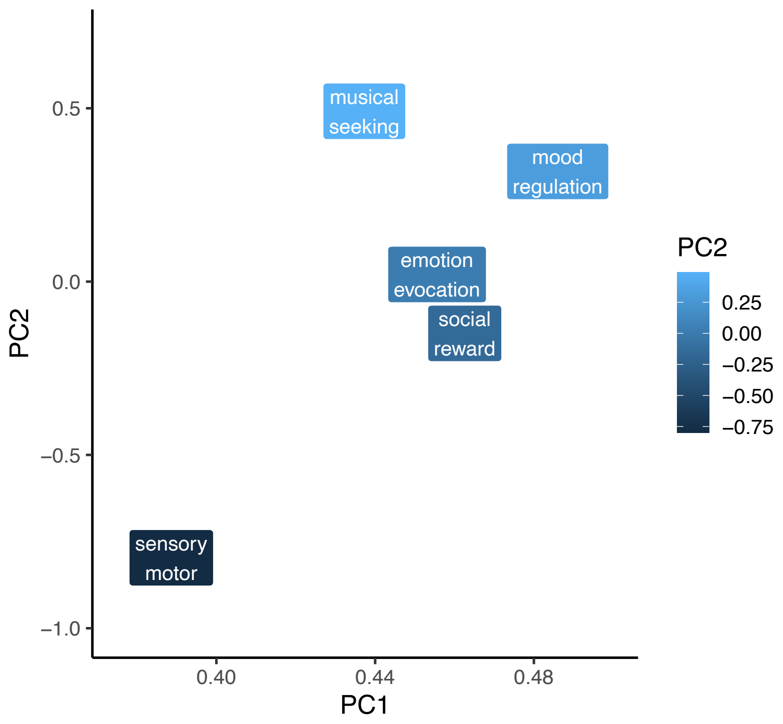

Supplementary Figure 11. *Principal component analysis of the additive genetic correlation matrix.* First and second principal component (PC) coordinates (i.e., Eigenvector elements 1 and 2) of the facets' additive genetic variances.

Supplementary Tables

Supplementary Table 1. *Mean and phenotypic standard deviations for the Swedish Musical Discrimination Test (SMDT), Behavioral Approach System Reward Responsiveness (BAS-RR), and Barcelona Music Reward Questionnaire (BMRQ) total scores and the five BMRQ facets scores.*

Measure	Descriptives				Effect size
	Women		Men		
	M	σ	*M*	σ	*d*
SMDT	44.26	7.33	45.47	7.99	-0.15
BAS-RR	16.54	2.23	15.98	2.23	0.25
BMRQ	76.18	13.41	71.10	14.10	0.37
emotion evocation	17.68	3.31	16.35	3.67	0.38
mood regulation	16.71	3.28	15.95	2.30	0.23
music seeking	12.14	3.80	12.93	3.91	-0.21
sensory motor	16.18	3.48	13.34	3.78	0.78
social reward	13.51	3.58	12.58	3.59	0.25

Note. Estimates are extracted from a zygosity model, which includes age as a covariate and constrains means (M) and standard deviations (σ) across twins of a pair, means and variances across sex-specific zygosity groups, and age variances across groups to be equal. Column *d* includes Cohen's *d* effect sizes for the differences between women and men means. SMDT: Swedish Music Discrimination Test; BAS-RR: Behavioural Approach System Reward Responsiveness; BMRQ: Barcelona Music Reward Questionnaire.

Supplementary Table 2. *Singleton and full-pairs means for the Swedish Musical Discrimination Test (SMDT), Behavioral Approach System Reward Responsiveness (BAS-RR), and Barcelona Music Reward Questionnaire (BMRQ) total scores and the five BMRQ facets scores.*

Measure		Sample		Test statistics		
	Sex	Singleton	Full-pairs	*t*	*df*	*p*
SMDT	**Women**	**39.36**	**40.61**	**-3.25**	**1062.2**	**.001**
	Men	40.40	42.06	-2.415	425.22	.020
BAS-RR	Women	16.05	15.95	1.35	3256.7	.177
	Men	15.54	15.35	1.64	1300.3	.102
BMRQ	Women	73.42	73.50	-0.17	3385	.867
	Men	67.25	68.49	-1.62	1366.1	.101
emotion evocation	Women	16.32	16.29	0.23	3397.1	.819
	Men	14.78	15.13	-1.86	1368	.063
mood regulation	Women	15.88	15.93	-0.42	3398.3	.675
	Men	14.97	15.19	-1.28	1364.9	.202
music seeking	Women	11.87	11.81	0.46	3382.3	.645
	Men	12.38	12.53	-0.69	1366.8	.488
sensory motor	Women	16.10	16.00	0.78	3390.5	.435
	Men	12.99	13.07	-0.43	1355.8	.671
social reward	Women	13.26	13.46	-1.70	3363.3	.089
	Men	12.14	12.54	-2.07	1362.4	.039

Note. The singleton column displays raw means for twins where data were only available from one twin in a pair, stratified by sex. The full-pairs column displays means for twins with data from the complete pair. Test statistics were obtained from Welch's two-sided two-sample t-test. SMDT: Swedish Music Discrimination Test; BAS-RR: Behavioural Approach System Reward Responsiveness; BMRQ: Barcelona Music Reward Questionnaire. The only significant difference is detected in women's SMDT scores, indicating that singleton women display lower music perceptual abilities than twins with a cooperative twin, indicating possible sources of selection bias. In **bold,** significant differences indicate possible sampling bias.

Supplementary Table 3. *Assumptions of equality of means and variances of the BMRQ sum score.*

Model	$\chi^2(df)$	AIC	BIC	*p*
Baseline covariate	3.04 (4)	120967	121247	.55
Twin order	14.03 (21)	120944	121108	.868
Zygosity	25.78 (29)	120940	121049	.637
Sex				
**Same mean**	**300.54 (30)**	**121212**	**121315**	**<.001**
Same variance	36.18 (30)	120948	121050	.202
Same covariances (MZ and DZ)	37.37 (32)	120945	121034	.236
Same covariances (DZ and DZos)	41.13 (33)	120947	121029	.156

Note. Model comparison test statistics for the assumption of equality of means and variances across zygosity and sex. Models are tested against the saturated model. Models are recursively nested, except for sex models, for which parameters are selectively constrained (e.g., 'Same variance' is not nested in the 'Same means' model since removing the covariate results in a deterioration of the overall fit). MZ: Monozygotic; DZ: Dizygotic; DZos: Dizygotic opposite-sex. In **bold**, the model with a significant deterioration of fit.

Supplementary Table 4. *Univariate ADE model comparison.*

Model	$\Delta\chi^2$ (Δdf)	AIC	BIC	*p*
ADE	41.13 (33)	120947	121022	.120
**AE**	**1.63 (1)**	**120947**	**121022**	**.201**
E	458.49 (1)	121471	121471	<.001

Note. Model comparison test statistics across univariate ADE models. ADE model tested against the saturated model. Other models are recursively nested and tested against the model appearing in the above row (i.e., χ^2(Δdf) are obtained in reference to the above models). The AE model is marked in **bold** as the most parsimonious mode

Supplementary Table 5. *Estimates for the path coefficient computed using the Cholesky approach.*

Path	*est*	*SE*	*est.std*	*SE.std*	$\Delta\chi^2$ (1)	*z*	*p*	p_{MLR}
λ_{A12}	-0.02	.01	-.05	.03	2.79	-1.57	.09	.114
**λ_{A13}**	**0.55**	**.06**	**.25**	**.02**	**94.38**	**9.82**	**<.001**	**<.001**
**λ_{A23}**	**3.30**	**.35**	**.31**	**.03**	**87.71**	**9.27**	**<.001**	**<.001**
λ_{E12}	0.04	.02	.08	.04	4.63	2.17	.03	.044
λ_{E13}	-0.01	.10	-.002	.03	0.00	-0.06	.95	.949
**λ_{E23}**	**1.125**	**.14**	**.15**	**.02**	**64.07**	**8.03**	**<.001**	**<.001**

Note. Path coefficient unstandardised (*est*) and standardised (*est.std*) estimates are reported in the second and fourth columns, respectively. Note that coefficients are standardised over the total phenotypic variance, including the effect of age. Standard errors (*SE*) are reported for the standardised path coefficients. Robust statistics are obtained from the "MLR" estimators, p_{MLR}. In **bold,** significant paths.

Supplementary Table 6. *BRMQ facets: univariate ADE and ACE model comparisons.*

Model	$\Delta\chi^2$ (1)	AIC	BIC	*p*
Emotion evocation				
ADE		95945	96027	
AE	2.37 (1)	95946	96021	.123
E	243.08 (1)	96187	96255	<.001
Mood regulation				
ADE		94974	95056	
AE	2.38 (1)	94974	95049	.123
E	286.00 (1)	95224	95293	<.001
Music seeking				
ADE		97773	97855	
AE	4.56 (1)	97776	97851	.033
E	323.12 (1)	98097	98165	<.001
Sensory motor				
ADE		96541	96623	
AE	5.71 (1)	96545	96623	.017
E	409.67 (1)	96953	97021	<.001
Social reward				
ACE		96380	96462	
AE	0.05 (1)	96378	96453	.82
E	447.52 (1)	96824	96892	<.001

Note. Model comparison test statistics across univariate ADE or ACE, AE, and E models. Models are recursively nested and tested against the full model. Note that model selection is based on $\alpha = .007$.

Supplementary Table 7. *Test statistic for multivariate model comparison.*

Model	$\Delta\chi^2$ (df)	AIC	BIC	*p*
AE distinct factor solution	537.007 (334)	276540	276889	<.001
AE shared-genetic factor solution	65.14 (5)	276596	276910	<.001
AE shared-environment factor solution	34.125 (5)	276565	276879	<.001

Note. Model comparison test statistics across multivariate models. Models are tested against the distinct factor solution.

Supplementary Table 8. *Differences between genetic or environmental correlations.*

Pair	Correlation	Δr	$\Delta\chi^2$ (1)	*p*	*z*	p_{MLR}
Emotion evocation						
**Mood regulation**	***Genetic (r_A)***	**0.10**	**7.89**	**.004**	**2.32**	**.002**
	Environmental (r_E)	-0.00	0.01	.92	-0.06	.95
**Music seeking**	r_A	**0.20**	**11.92**	**<.001**	**3.95**	**<.001**
	r_E	-0.07	1.82	.18	-1.34	.18
Sensory motor	r_A	0.12	3.59	.06	2.68	.007
	r_E	-0.04	0.81	.37	-0.87	.39
**Social reward**	r_A	**-0.18**	**28.716**	**<.001**	**-3.89**	**<.001**
	r_E	-0.05	1.04	.31	-1.03	.30
Mood regulation						
Music seeking	r_A	0.10	2.41	.12	2.46	.01
	r_E	-0.07	0.98	.32	-1.44	.15
Sensory motor	r_A	0.03	0.18	.67	0.68	.50
	r_E	-0.04	2.18	.14	-0.81	.41
**Social reward**	r_A	**-0.28**	**66.425**	**<.001**	**-6.14**	**<.001**
	r_E	-0.05	0.70	.40	-0.99	.32
Music seeking						
Sensory motor	r_A	-0.07	2.75	.10	-1.74	.08
	r_E	0.03	0.30	.58	0.52	.60
**Social reward**	r_A	**-0.39**	**76.81**	**<.001**	**-8,20**	**<.001**
	r_E	0.02	0.01	.92	0.41	.68
Sensory motor						
**Social reward**	r_A	**-0.31**	**50.81**	**<.001**	**-6.86**	**<.001**
	r_E	-0.01	0.01	.92	-0.11	.91

Note. The differences between correlations were obtained by subtracting Fisher-z transformed r values. For ease of interpretation, *r* back-transformed values of the differences are reported (Δr). *p*: p-value obtained by LRT; p_{MLR}: p-value obtained via z-test, model fit using maximum likelihood with sandwich (Huber-White) standard error; in **bold**, covariances that could not be constrained to be equal.

Supplementary References

1. Yuan, K.-H. & Bentler, P. M. 5. Three Likelihood-Based Methods for Mean and Covariance Structure Analysis with Nonnormal Missing Data. *Sociol. Methodol.* **30**, 165–200 (2000).
2. Rosseel, Y. lavaan: An R Package for Structural Equation Modeling. *J. Stat. Softw.* **48**, 1–36 (2012).
3. de Hoyos, L. *et al.* Structural models of genome-wide covariance identify multiple common dimensions in autism. *Nat. Commun.* **15**, 1770 (2024).
4. Mas-Herrero, E., Zatorre, R. J., Rodriguez-Fornells, A. & Marco-Pallarés, J. Dissociation between Musical and Monetary Reward Responses in Specific Musical Anhedonia. *Curr. Biol.* **24**, 699–704 (2014).
5. Martínez-Molina, N., Mas-Herrero, E., Rodríguez-Fornells, A., Zatorre, R. J. & Marco-Pallarés, J. Neural correlates of specific musical anhedonia. *Proc. Natl. Acad. Sci.* **113**, E7337–E7345 (2016).
6. Martínez-Molina, N., Mas-Herrero, E., Rodríguez-Fornells, A., Zatorre, R. J. & Marco-Pallarés, J. White Matter Microstructure Reflects Individual Differences in Music Reward Sensitivity. *J. Neurosci.* **39**, 5018–5027 (2019).
7. Hernández, M. *et al.* Separate Contribution of Striatum Volume and Pitch Discrimination to Individual Differences in Music Reward. *Psychol. Sci.* **30**, 1352–1361 (2019).
8. Belfi, A. M. & Loui, P. Musical anhedonia and rewards of music listening: current advances and a proposed model. *Ann. N. Y. Acad. Sci.* **1464**, 99–114 (2020).
9. Zatorre, R. J. & Salimpoor, V. N. From perception to pleasure: Music and its neural substrates. *Proc. Natl. Acad. Sci.* **110**, 10430–10437 (2013).
10. Zatorre, R. *From Perception to Pleasure: The Neuroscience of Music and Why We Love It*. (Oxford University Press, 2023). doi:10.1093/oso/9780197558287.001.0001.
11. Mas-Herrero, E., Marco-Pallares, J., Lorenzo-Seva, U., Zatorre, R. J. & Rodriguez-Fornells, A. Individual Differences in Music Reward Experiences. *Music Percept.* **31**, 118–138 (2013).
12. Carver, C. S. & White, T. L. Behavioral inhibition, behavioral activation, and affective responses to impending reward and punishment: The BIS/BAS Scales. *J. Pers. Soc. Psychol.* **67**, 319–333 (1994).
13. Eriksson, L. J. K., Jansson, B. & Sundin, Ö. Psychometric properties of a Swedish version of the reinforcement sensitivity theory of personality questionnaire. *Nord. Psychol.* **71**, 134–145 (2019).
