# Supplementary material for: Twin modelling reveals partly distinct genetic pathways to music enjoyment
Source: Nat Commun. 2025 Mar 25;16:2904. doi: 10.1038/s41467-025-58123-8 (PMC11937235; doi:10.1038/s41467-025-58123-8)
Supplement: Supplementary file 2 — Reporting Summary [file 41467_2025_58123_MOESM2_ESM.pdf]

nature portfolio

Corresponding author(s): Giacomo Bignardi
Last updated by author(s): Dec 2, 2024

Reporting Summary

Nature Portfolio wishes to improve the reproducibility of the work that we publish. This form provides structure for consistency and transparency in reporting. For further information on Nature Portfolio policies, see our Editorial Policies and the Editorial Policy Checklist.

Statistics

For all statistical analyses, confirm that the following items are present in the figure legend, table legend, main text, or Methods section.

n/a	Confirmed	
☐	☒	The exact sample size (*n*) for each experimental group/condition, given as a discrete number and unit of measurement
☐	☒	A statement on whether measurements were taken from distinct samples or whether the same sample was measured repeatedly
☐	☒	The statistical test(s) used AND whether they are one- or two-sided<br>*Only common tests should be described solely by name; describe more complex techniques in the Methods section.*
☐	☒	A description of all covariates tested
☐	☒	A description of any assumptions or corrections, such as tests of normality and adjustment for multiple comparisons
☐	☒	A full description of the statistical parameters including central tendency (e.g. means) or other basic estimates (e.g. regression coefficient) AND variation (e.g. standard deviation) or associated estimates of uncertainty (e.g. confidence intervals)
☐	☒	For null hypothesis testing, the test statistic (e.g. *F*, *t*, *r*) with confidence intervals, effect sizes, degrees of freedom and *P* value noted<br>*Give P values as exact values whenever suitable.*
☒	☐	For Bayesian analysis, information on the choice of priors and Markov chain Monte Carlo settings
☐	☒	For hierarchical and complex designs, identification of the appropriate level for tests and full reporting of outcomes
☐	☒	Estimates of effect sizes (e.g. Cohen's *d*, Pearson's *r*), indicating how they were calculated

Our web collection on statistics for biologists contains articles on many of the points above.

Software and code

Policy information about availability of computer code

Data collection	*Provide a description of all commercial, open source and custom code used to collect the data in this study, specifying the version used OR state that no software was used.*
Data analysis	Data were analyzed using R. The following packages were used for main data analysis:tidyverse, rstatix, psych, and lavaan. Figures were made by making use of the following packages: ggplot and patchwork;Twin models were specified using costumed code in lavaantwda, available at: https://github.com/giacomobignardi/h2_BMRQ/tree/main/R/functions/lavaantwda.

For manuscripts utilizing custom algorithms or software that are central to the research but not yet described in published literature, software must be made available to editors and reviewers. We strongly encourage code deposition in a community repository (e.g. GitHub). See the Nature Portfolio guidelines for submitting code & software for further information.

Data

Policy information about availability of data

All manuscripts must include a data availability statement. This statement should provide the following information, where applicable:

- Accession codes, unique identifiers, or web links for publicly available datasets
- A description of any restrictions on data availability
- For clinical datasets or third party data, please ensure that the statement adheres to our policy

The datasets generated during the current study cannot be made public as registry data were used. However, researchers are able to apply online at the Swedish Twin Registry to access the twin data used in this study (see https://ki.se/en/research/swedish-twin-registry-for-researchers).

Research involving human participants, their data, or biological material

Policy information about studies with human participants or human data. See also policy information about sex, gender (identity/presentation), and sexual orientation and race, ethnicity and racism.

Reporting on sex and gender	In the main and supplementary material we report descriptives mean differences in music perceptual abilities, general reward sensitivity, and music reward sensitivity (and its facets). Findings apply to participants self-reported sex only.
Reporting on race, ethnicity, or other socially relevant groupings	We did not consider additional groupings in our analysis.
Population characteristics	Swedish twins of mean age of 51 years (sd = 8 years, range from 37 to 64 years).
Recruitment	Participants were twins recruited from the Swedish Twin Registry. The twins included in this study were part of the Study of Twin Adults: Genes and Environment (STAGE) cohort. The STAGE cohort is a sample of twins born in Sweden between 1959 and 1985. The twins included in this study took part in two large recent waves of online data collection on music, art and cultural engagement (the Humans Making Music study). In 2011 and then again in 2022, a total of 32,005 adult twin individuals from the STAGE cohort were invited to participate in an online-based study. Participants took the Swedish Musical Discrimination Test in the first wave. Additionally, in the second wave of data collection, we collected novel data using the Behavioral Approach System (BAS) and Barcelona Music Reward Questionnaire (BMRQ).
Ethics oversight	Both studies were approved by the Regional Ethical Review Board in Stockholm (Dnrs 2011/570-31/5, 2012/1107/32, 2021-02014, 2022-00109-02, 2020-02575).

Note that full information on the approval of the study protocol must also be provided in the manuscript.

Field-specific reporting

Please select the one below that is the best fit for your research. If you are not sure, read the appropriate sections before making your selection.

☐ Life sciences ☒ Behavioural & social sciences ☐ Ecological, evolutionary & environmental sciences

For a reference copy of the document with all sections, see nature.com/documents/nr-reporting-summary-flat.pdf

Behavioural & social sciences study design

All studies must disclose on these points even when the disclosure is negative.

Study description	Quantitative approach; Classical Twin Design.
Research sample	Population-based sample of same-sex and opposite-sex twin pairs and single twins recruited via the Swedish Twin Registry.
Sampling strategy	The entire twin cohort was invited for participation and everyone who participated in the latest wave was included in the present study.
Data collection	Data were collected online. All twins of the respective cohort were invited via a letter send to their home. The twins were asked to log-in online (after verifying their personal ID via Bank-ID) and fill out the questionnaire targeted mostly at musical and leisure engagement and skills among personality and some mental health related measures. For the collection of the main variables of interest to this study in 2022, three reminders were send out to the twins. Participation was reimbursed with a 100 SEK shopping voucher.
Timing	Data were collected in 2011/2012 (collection of musical discrimination ability) and again in 2022/2023 (collection of main variable of interest).
Data exclusions	Only individuals with available BMRQ data were included in the study. No other data were excluded from analysis.
Non-participation	Of the total of 32,005 adult twin individuals (first wave) around 11,500 participated in the first wave and then around 9,500 in the

	latest wave. We note response rates in the STAGE cohort have been low. The low response rates are owed to the fact that this is a population-based study (i.e., we have invited a whole birth cohort of Swedish twins to participate in our studies) of a working-aged cohort and is a general phenomenon not unique to our sample/study.
Randomization	n/a

Reporting for specific materials, systems and methods

We require information from authors about some types of materials, experimental systems and methods used in many studies. Here, indicate whether each material, system or method listed is relevant to your study. If you are not sure if a list item applies to your research, read the appropriate section before selecting a response.

Materials & experimental systems

n/a	Involved in the study	
☒	☐	Antibodies
☒	☐	Eukaryotic cell lines
☒	☐	Palaeontology and archaeology
☒	☐	Animals and other organisms
☒	☐	Clinical data
☒	☐	Dual use research of concern
☒	☐	Plants

Methods

n/a	Involved in the study	
☒	☐	ChIP-seq
☒	☐	Flow cytometry
☒	☐	MRI-based neuroimaging

Plants

Seed stocks	*Report on the source of all seed stocks or other plant material used. If applicable, state the seed stock centre and catalogue number. If plant specimens were collected from the field, describe the collection location, date and sampling procedures.*
Novel plant genotypes	*Describe the methods by which all novel plant genotypes were produced. This includes those generated by transgenic approaches, gene editing, chemical/radiation-based mutagenesis and hybridization. For transgenic lines, describe the transformation method, the number of independent lines analyzed and the generation upon which experiments were performed. For gene-edited lines, describe the editor used, the endogenous sequence targeted for editing, the targeting guide RNA sequence (if applicable) and how the editor was applied.*
Authentication	*Describe any authentication procedures for each seed stock used or novel genotype generated. Describe any experiments used to assess the effect of a mutation and, where applicable, how potential secondary effects (e.g. second site T-DNA insertions, mosiacism, off-target gene editing) were examined.*
